# Chromatographic Methods for the Determination of Glyphosate in Cereals Together with a Discussion of Its Occurrence, Accumulation, Fate, Degradation, and Regulatory Status

**DOI:** 10.3390/mps7030038

**Published:** 2024-05-02

**Authors:** Maurizio Masci, Roberto Caproni, Teresina Nevigato

**Affiliations:** Council for Agricultural Research and Economics (CREA), Research Centre for Food and Nutrition, via Ardeatina 546, 00178 Rome, Italyteresina.nevigato@crea.gov.it (T.N.)

**Keywords:** glyphosate, analytical methods, sample preparation, liquid chromatography, gas chromatography, mass spectrometry, cereals, occurrence, accumulation mechanism, regulatory status

## Abstract

The European Union’s recent decision to renew the authorization for the use of glyphosate until 15 December 2033 has stimulated scientific discussion all around the world regarding its toxicity or otherwise for humans. Glyphosate is a chemical of which millions of tons have been used in the last 50 years worldwide to dry out weeds in cultivated fields and greenhouses and on roadsides. Concern has been raised in many areas about its possible presence in the food chain and its consequent adverse effects on health. Both aspects that argue in favor of toxicity and those that instead may indicate limited toxicity of glyphosate are discussed here. The widespread debate that has been generated requires further investigations and field measurements to understand glyphosate’s fate once dispersed in the environment and its concentration in the food chain. Hence, there is a need for validated analytical methods that are available to analysts in the field. In the present review, methods for the analytical determination of glyphosate and its main metabolite, AMPA, are discussed, with a specific focus on chromatographic techniques applied to cereal products. The experimental procedures are explained in detail, including the cleanup, derivatization, and instrumental conditions, to give the laboratories involved enough information to proceed with the implementation of this line of analysis. The prevalent chromatographic methods used are LC-MS/MS, GC-MS/SIM, and GC-MS/MS, but sufficient indications are also given to those laboratories that wish to use the better performing high-resolution MS or the simpler HPLC-FLD, HPLC-UV, GC-NPD, and GC-FPD techniques for screening purposes. The concentrations of glyphosate from the literature measured in wheat, corn, barley, rye, oats, soybean, and cereal-based foods are reported, together with its regulatory status in various parts of the world and its accumulation mechanism. As for its accumulation in cereals, the available data show that glyphosate tends to accumulate more in wholemeal flours than in refined ones, that its concentration in the product strictly depends on the treatment period (the closer it is to the time of harvesting, the higher the concentration), and that in cold climates, the herbicide tends to persist in the soil for a long time.

## 1. Introduction

**Figure 1:**
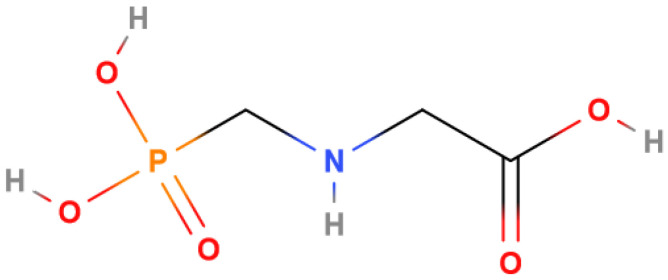
Glyphosate.

Glyphosate, with the IUPAC name N-(phosphonomethyl)glycine, also called 2-(phosphonomethylamino)acetic acid (Figure 1), is the most used herbicide in the world. It was introduced to the market in 1974 under the trade name of Roundup^®^ for weed control in agriculture or for eliminating roadside weeds, as well as in orchards, forests, parks, and squares, and on railways. In traditional agriculture, glyphosate was used only in the pre-emergence phase, but after the introduction of genetically modified organisms (GMOs) resistant to Roundup^®^, it also began to be used in the post-emergence phase. In 1996, genetically modified soybean, corn, and cotton plants, called ‘Roundup^®^ Ready’ plants, were introduced onto the market. Since then, the use of genetically modified organisms and the use of glyphosate have grown to unprecedented levels: about 660 million kg of global glyphosate use in 2011 and 826 million in 2014 are reported [1,2,3].

However, the improper use of this herbicide in the pre-harvest phase gave rise to a heated debate. This controversial application method involves applying it directly to the crop a short time before harvesting for drying purposes, to optimize yields [3,4]. This happens especially in climates that are not fully suitable for some cultivations. In the US, in Canada, and elsewhere, there is a practice of using glyphosate to desiccate crops by spraying the maturing plants, in order to speed up the ‘maturation’ of the crop and make it more uniform, thereby facilitating harvest [5,6,7,8,9,10,11,12]. This may add to the residue levels of glyphosate, as shown in field pea, barley, and flax seed. Particularly if the plant is still growing, translocation of the herbicide within the plant may result in its accumulation in the seed, both for GM and unmodified soy [13]. Even Mediterranean countries import significant quantities of wheat from those countries that use glyphosate in the pre-harvest phase [14]. Plants translocate this systemic herbicide to their roots, shoots, seeds, and fruits, where it causes the accumulation of shikimic acid and hinders the enzymatic conversion of shikimic acid to anthranilic acid by inhibiting the enzyme 5-enolpyruvylshikimic acid-3-phosphate synthase [15,16,17]. Crops treated with glyphosate slowly die over a period of days or weeks, and because the chemical is transported throughout the plant, no part survives [18]. Because plants absorb glyphosate, it cannot be completely removed by washing or peeling produce or by milling, baking, or brewing grains [1], although in this way, its content can be somewhat reduced [14]. In the environment, the free pesticide degrades rapidly, but when it comes into contact with the soil, it adsorbs to soil particles and degrades very slowly; sometimes, it remains undegraded and inactive in the soil for years [18,19], dissociating only into its main degradation product [20], namely aminomethylphosphonic acid (AMPA), which has comparable toxicity to glyphosate and which must always be analytically determined together with the latter.

In 2017, the IARC, the International Agency for Research on Cancer, classified this pesticide in Group 2A, ‘probably carcinogenic to humans’ [21]. On the contrary, the European Commission in 2016 established that ‘on the basis of the information currently available, no hazard classification for carcinogenicity is justified for glyphosate’ [22], and therefore, in December 2017, it renewed the authorization for its use until 2022 [22,23]. In 2022, the decision about the use of glyphosate was postponed, and in November 2023, the Commission authorized the use of glyphosate as a herbicide until 2033 [24], also based on an opinion delivered by the European Food Safety Authority (EFSA) [25]. Some studies declare that glyphosate is toxic. New research indicates that glyphosate causes leukemia in the early life of rats administered the herbicide via drinking water, at doses currently considered safe by regulatory agencies [26]. The same considered-safe doses showed endocrine toxicity in rats [27], later confirmed in a human population of mothers and newborns exposed to glyphosate during pregnancy [28]. A very recent review of Lacroix and Kurrasch is less conclusive. They observe that co-formulants in Glyphosate preparations can greatly amplify toxicity; indeed, the co-formulants themselves may be more toxic than Glyphosate itself [29]. Therefore, the question of toxicity remains open.

In this context, the availability of reliable analytical methods is crucial. The highly topical debate on a herbicide declared in the past to be of little danger currently requires more careful evaluations and a greater number of analytical measurements to understand its fate once used in the field and how much of it passes into the various final products intended for consumption, with the consequent degree of exposure for consumers. Due to some of its molecular characteristics (the absence of UV absorbance, low volatility, and high hydrophilicity) [1,30,31,32,33], the quantitative determination of glyphosate in crops, in soils, and in waters is challenging. Chromatography is the most successful and the most used technique, which, very frequently, exploits derivatization (mandatory in gas chromatography). When liquid chromatography with derivatization is applied, the prevalent derivatizing agent is FMOC-Cl (9-fluorenylmethyl chloroformate). FMOC-Cl reacts with glyphosate and AMPA to give the corresponding derivatives [33,34,35,36,37,38,39,40,41,42]. The determination of glyphosate by liquid chromatography is also possible without derivatization. As regards gas chromatography (GC), a largely used derivatization is that of using perfluoroalcohols plus trifluoroacetic anhydride [43,44,45,46,47,48,49,50,51,52]. The perfluoroalcohol used is trifluoroethanol (TFE) or heptafluorobutanol (HFB). In addition, another GC derivation method exists, which is used to a lesser extent: alkylsilyl derivatization. In the present review, all methods for determining glyphosate and AMPA in cereals via liquid and gas chromatography will be discussed in detail.

## 2. Glyphosate Degradation Pathways

**Figure 2:**
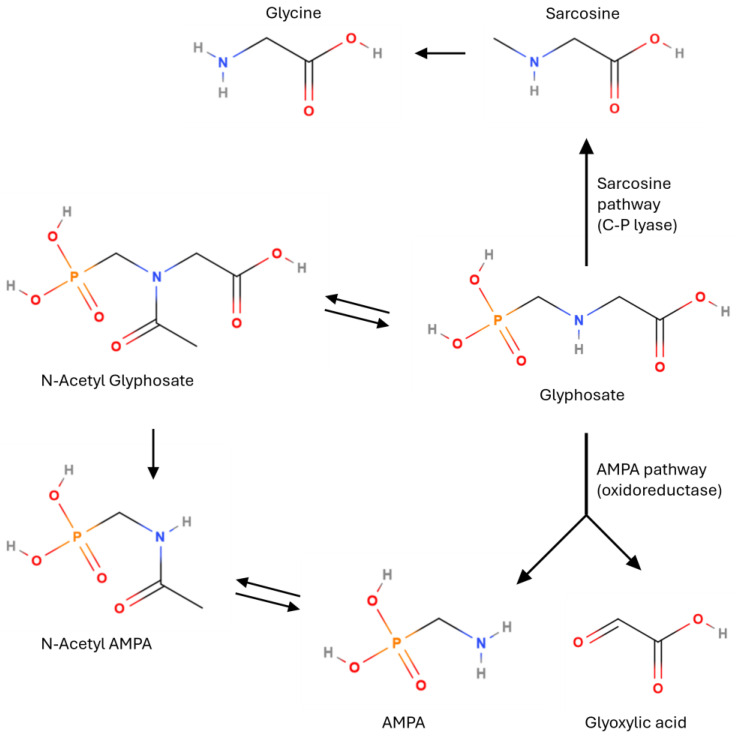
The two main degradation pathways of glyphosate.

In general, glyphosate degradation proceeds by either of two pathways [53,54,55,56] as shown in Figure 2. They are also called ‘AMPA pathway’ and ‘sarcosine pathway’.

Glyphosate is either transformed into AMPA and glyoxylic acid by oxidoreductase or into sarcosine by C-P lyase [57]. Sarcosine is then converted to glycine by sarcosine oxidase [55], while glyoxylic acid, in turn, is converted to glycine and carbon dioxide by the glyoxylic acid cycle [53,55]. AMPA is presumed to be converted to methylamine [54]. Among the degradation products of glyphosate, AMPA is the only persistent compound. From an analytical point of view, it is of primary importance to always monitor AMPA together with glyphosate since AMPA is the main metabolite of glyphosate [58,59]; from some research, it appears to have equal or greater toxicity compared to glyphosate itself [59,60]. The degradation of AMPA is generally slower than that of glyphosate, possibly because AMPA may adsorb onto soil particles more strongly than glyphosate and/or because it may be less likely to permeate the cell walls or membranes of soil microorganisms [55]. The conversion pathway to N-Acetyl-derivatives in Figure 2 is typical of genetically modified (GM) plants. After glyphosate is applied to GM soybean, the metabolite N-Acetyl glyphosate is formed [55]. According to the U.S. Environmental Protection Agency, its toxicological effects are similar to those of glyphosate, while in pursuance of EFSA data, there is a lack of studies for N-acetyl-glyphosate’s and N-acetyl-AMPA’s toxicological effects [55]. According to the EFSA’s opinion about maximum residue levels, glyphosate is considered to be a sufficient marker for conventional crops, while for plants with glyphosate-tolerant GM varieties, N-Acetyl glyphosate should also be determined.

## 3. Occurrence Data, Accumulation, and Fate

Table 1 shows an overview of the glyphosate and AMPA levels measured in cereals.

### 3.1. Glyphosate Concentration in Straight-Grade Flour vs. Concentration in Wholemeal Flour

It was observed that glyphosate is more present in bran and less present in white flour. Granby et al. sampled grains from Danish mills and major producers in 1998–2001 and carried out an analysis of glyphosate residues. The average results for wheat showed that the glyphosate content in bran was concentrated compared with the grain, while its content in flour was somewhat lower than in grain [61]. It may be reasonable to further consider that glyphosate is generally sprayed directly onto the crops and that the extractable glyphosate is then directly correlated with the presence of the outer bran. Reasonably, the differences in glyphosate in whole wheat where the bran remains and the refined flours where the bran is removed may explain the differences observed in extractable glyphosate in commercially available food samples [71]. Tittlemier et al. demonstrated that 50% of the total glyphosate mass resides in the outer 17% of the kernels and that 81% of the total glyphosate mass in wheat was associated with the bran, shorts, and feeds milling fractions. They argue that glyphosate concentrations in bread made from straight-grade flour will be approximately 4× lower than that made from whole-grain flour [72]. The results of a further one-year study showed that glyphosate residues were more concentrated on top of grain/seed layers, rather than inside, and that the highest concentrations of glyphosate residues were found in bran [5]. Other studies confirm such a trend [4,64,73,74] with Ashley-Martin et al. observing a dose–response relationship between the consumption of whole-grain bread and higher urinary glyphosate concentrations [75]. Obviously, crops that are grown without the use of glyphosate do not show any trace of residue in any part of the grain, not even in the bran [5,13]: this last statement is important for the recognized beneficial health effects related to the consumption of whole grains [76,77,78,79,80].

### 3.2. Glyphosate Accumulation in Crops

The period of crop treatment with the herbicide is critical to the concentration of glyphosate in the final harvested product.

Gélinas et al. applied Roundup^®^ before harvest to some wheat varieties in an amount of 0.82 kg ha^−1^, as recommended on the label, and observed a glyphosate residue of 11.1 mg kg^−1^ in one wheat variety and 6.1 mg kg^−1^ in another variety. This high residue content was attributed to the date of treatment, very close to the harvest time [4]. Bøhn et al. reported that GM soybean treated with Roundup^®^ during the growing season accumulated glyphosate and AMPA at concentrations of 0.4–8.8 mg kg^−1^ and 0.7–10 mg kg^−1^. In contrast, conventional and organic soybean crops did not contain glyphosate and AMPA [13].

**Figure 3:**
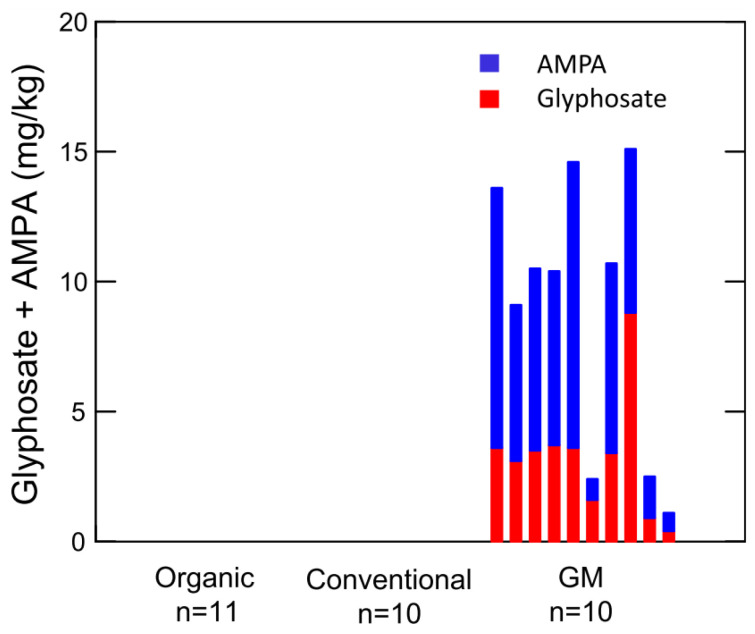
Residues of glyphosate and AMPA in individual soybean samples (*n* = 31). Excerpted from the work of Bøhn et al., 2014 [13].

Therefore, the authors confirmed the hypothesis that GM soybeans may contain high residue levels of glyphosate and AMPA due to repeated spraying of the plants with glyphosate-based herbicides throughout the production season (Figure 3).

Kadžienė et al. applied a study protocol to test for glyphosate accumulation in cereals following a pre-emergence treatment (1 week after sowing) and a pre-harvest treatment (14–10 days before harvest). From their study, repeated for two seasons, it was found that the pre-emergence application had no impact on the final contamination of the cereals (no residues). Instead, pre-harvest application resulted in maximum concentrations of 2.15 mg kg^−1^ for glyphosate and 0.04 mg kg^−1^ for AMPA [5].

### 3.3. Fate and Degradation of Glyphosate

Studies carried out in crops, and in products derived from them, indicate that washing wheat grains is able to reduce the concentration of glyphosate. Also, grain processing can reduce the concentration of the herbicide [71], as can decortication, i.e., the removal of the external layers of the grains, although glyphosate cannot be eliminated completely as it is absorbed by the entire plant. It was demonstrated that the preparation of dried pasta from semolina and flour milling fractions reduced concentrations of glyphosate by a factor of 1.8 [73]. In the same research, the concentrations in cooked pasta decreased significantly with cooking time: after 15 min of cooking, approximately 73% of the total glyphosate mass had transferred from the pasta to the cooking water [73]. The malting of barley results in the gross loss of residues: glyphosate residue levels in beer were found to be about 4% of the original levels in barley [74].

Conflicting results are available on glyphosate degradation resulting from dough fermentation during the breadmaking process [4,81]. Regarding storage, it was found that when glyphosate was applied during the pre-harvest period at the rate indicated by the label, its level in grain remained steady during a 2-year storage time [5].

Given the hydrophilicity of glyphosate, water bodies adjacent to a treated field are frequently polluted by glyphosate and AMPA [82,83,84,85,86], with associated possible human diseases [87]. Pires et al. observed that, with a few exceptions, glyphosate concentration in water samples was higher than AMPA, at a ratio that could reach almost 30 for groundwater samples, much higher than in surface samples (highest ratio of 3.6). This is probably because glyphosate in groundwater is not susceptible to photodegradation, an important degradation pathway in the environment [88]. Silva et al. measured the proportion of AMPA to glyphosate in agricultural topsoils of the European Union, deducing that soils presenting the lowest proportion of AMPA suggest more recent glyphosate applications and/or slower degradation of glyphosate into AMPA in those conditions [89]. For food matrices, especially processed ones, the glyphosate/AMPA ratio depends on too many variables. For example, in honey, this ratio does not show a constant value or any observable trend [90]. The dissipation rates of the herbicide in the field are influenced by the soil properties, application methods, and environmental conditions. Greater persistence has been observed in colder climates [91]: in northern climates with seasonally frozen soils, field studies have shown clear persistence of glyphosate throughout the winter. After applications in June and July at two Finnish sites, approximately 10–20% of the applied glyphosate was detected in June of the following year, demonstrating that the time for dissipation of 90% of glyphosate (DT 90) was about 11 months. In warmer climates, glyphosate did not persist beyond the growing season, even after 15 consecutive annual applications [92].

## 4. Regulatory Status

The toxicity of glyphosate for human health is still under study, and this can be deduced from Table 2. In the case of wheat, for example, FAO/WHO and EPA have set the MRL for glyphosate at 30 mg kg^−1^, while the European Union and Health Canada have set it 10 and 5 mg kg^−1^, respectively. Considering the controversy on glyphosate’s maximum residue level in foodstuffs [1], there is a possibility that the current MRLs could be modified in the coming years; future research will have to take into consideration even very low levels of intake and follow chronic exposures, to ascertain the possible negative effect of this herbicide on health.

## 5. Chemical Analytical Methods

Choosing an analytical method depends on the objective to be achieved. For compliance control purposes, an instrument with sensitivity of at least 1 mg kg^−1^ would be suitable for 91% of the established MRLs listed in Table 2. So, the much simpler and historically widely used HPLC–fluorimetry (HPLC-FLD) [98,99,100,101] or HPLC-UV [39,42,85] would be still suitable for the aim, in fact the limit of quantitation of these techniques is about 0.5 mg kg^−1^ [98]. Similar considerations apply to gas chromatography with nitrogen phosphorus (NPD) or a flame photometric detector (FPD), which are less specific detectors than mass spectrometers but of higher affordability and of good sensitivity [51,102]. However, for research work in complex matrices, more performing techniques are preferred. Currently there is a tendency to use the mass spectrometer as a chromatographic detector for every purpose given its high sensitivity and specificity.

### 5.1. Liquid Chromatography

**Figure 4:**
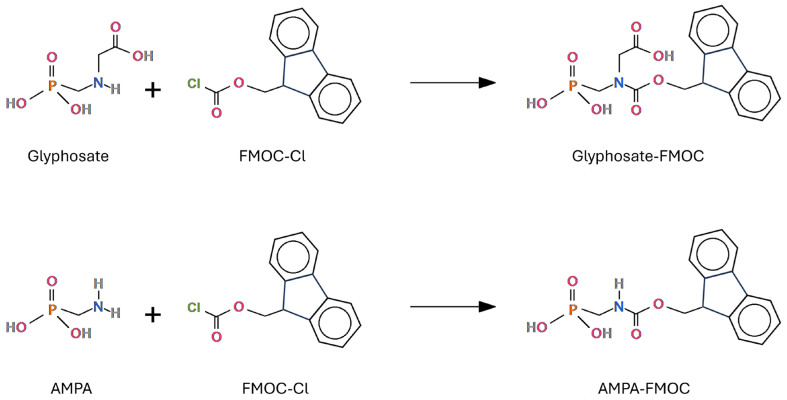
FMOC derivatization of glyphosate and AMPA for determinations carried out by liquid chromatography.

Analyses of glyphosate and AMPA in water have been performed for a long time by liquid chromatography with FMOC derivatization, and subsequently also codified by the ISO 16308:2014 standard [103,104]. The ISO 16308:2014 standard involves the use of a tandem mass spectrometer as a detector, but the method was originally based on HPLC–fluorimetry. By switching to mass spectrometry as a detection technique, better sensitivity was obtained. This method uses the derivatization of both molecules with 9-fluorenylmethyl chloroformate (FMOC-Cl), as shown in Figure 4.

This is needed due to the high polarity of the compounds as such, which would make analysis using reversed-phase liquid chromatography difficult. From the analysis of water, the liquid chromatography method via FMOC derivatization was quickly extended to the analysis of foods. In a 2015 interlaboratory ring test for the analysis of glyphosate in wheat flour, all the laboratories that used liquid chromatography with derivatization exploited FMOC derivatization, and almost all used tandem mass spectrometry (LC-MS/MS) as the detection technique [1]. It must be said that out of 12 laboratories, 5 did not use derivatization. The performance of the laboratories that participated in the study indicates that LC-MS/MS has a sensitivity of 0.03–0.05 mg per kg of product. Some recent works claim even better performance [35,62].

#### 5.1.1. FMOC Derivatization

Pre-column FMOC-Cl derivatization has been proven to be simple and successful. The complete reaction of glyphosate with FMOC-Cl guarantees stability and successful chromatographic separation on reversed-phase LC columns [33]. The derivatization takes place at an alkaline pH: generally, the pH is adjusted to 9 by adding borate buffer and by adding EDTA. With LC-MS/MS, the parent ions to be monitored are 390 *m/z* for glyphosate-FMOC and 332 *m/z* for AMPA-FMOC [33,34]. The daughter ions are frequently 150 *m/z* for glyphosate-FMOC and 110 *m/z* for AMPA-FMOC, so the transitions of interest are generally 390 → 150 *m/z* and 332 → 110 *m/z* [34,38], but also 168 → 63 *m/z* and 110 → 63 *m/z* in those cases where derivatization is not used [62]. Detection can be carried out in positive electrospray mode [36] or in negative electrospray ionization mode [33,34,38,62]. The eluting solvents are often ammonium acetate in LC−MS-grade water, and acetonitrile (ACN). Martin et al. evaluated the influence of borate addition to the derivative step and the responses of various borate buffer concentrations (*w*/*v*). They found that the reaction did not occur in the control, for which no borate buffer was added, while when borate buffer was added to the reaction medium, the peak intensities of the derivative products were greater: a significantly higher response was obtained with 5% borate addition, compared to the control, than the other variables [33]. The reactivity of glyphosate’s amino group was enhanced by increasing the buffer concentration, which improved the derivatization reagent’s solubility.

Before carrying out the derivatization step, the extraction of glyphosate and AMPA from the sample must be performed. In the next section, an overview of sample preparation will be given with some practical examples.

#### 5.1.2. Sample Preparation Used with FMOC Derivatization

A good practical example of the analysis of glyphosate in cereals via liquid chromatography is the work conducted in 2021 by Cruz and Murray from the U.S. National Institute of Standards and Technology (NIST), who analyzed oat products (oatmeal, oat-based cereals, and oat flour, both conventional and organic). They used a Solid Phase Extraction (SPE) step. To the ground cereal sample (1 g), internal standard aqueous solution was added (isotopically labeled glyphosate and AMPA); then, an aliquot of a 50/50 mix of acidified H_2_O (0.1% formic acid) and MeOH was added, and a centrifugation step followed. After centrifuging, the sample was transferred into a polypropylene centrifuge tube containing borate buffer reagent. After homogenization, FMOC reagent was added, and the sample was left in the dark during the derivatization reaction. Then, the SPE step was carried out by adjusting the pH, centrifugating, loading the supernatant onto the SPE cartridge, and eluting with a MeOH/NH_4_OH solution mix in a 2 mL glass vial. The eluate was evaporated, and H_2_O was added to obtain a final volume of 500 µL. The extracts were filtered and stored at 6 °C until LC–MS/MS analysis. Separation was carried out on a C18 column, where the injection volume was 20 µL and the monitored transitions for quantification were 390 → 168 *m/z* for glyphosate-FMOC and 332 → 110 *m/z* for AMPA-FMOC [105].

Similar preparation was used for soy protein isolate [36], cereal flour samples by means of HPLC-FLD [98], tea samples [34], and beebread samples [38].

From the examination of the works cited, it can be deduced that a cleanup step via SPE is always used for the determination of glyphosate and AMPA in food matrices such as cereals, which present a certain complexity (while it is not necessary for the sample preparation of drinking water or groundwater, for example, which are quite clean themselves). The SPE technique allows the enrichment and purification of target analytes and is a decisive step before injecting the sample into modern analytical instruments capable of exceptional sensitivity. The basic principles of SPE are similar to liquid–liquid extraction, but SPE involves the dispersion of the analyte between a liquid and a solid phase. A commonly used procedure includes conditioning of the SPE cartridge with solvents. Then, the extracted sample solution is loaded onto the cartridge. Elution is carried out with a suitable solvent and the effluent is collected for the next step. SPE materials (carbon, silica, clay, resins) are available as tubes or cartridges and are commercially known by various names. One of the most used is Oasis^®^ HLB from Waters (Milford, MA, USA) [34,35,38], but the following are also widespread: Oasis^®^ WCX [34], Oasis^®^ MAX [34], Oasis^®^ MCX, C18 sorbents, ion exchange BondElut Plexa PAX^®^ (Agilent, Santa Clara, CA, USA), InertSep SAX^®^ (GL Sciences, Tokyo, Japan), Extrelut, Florisil, Chromabond^®^ (Macherey-Nägel, Hœrdt, France) [41], and others [3].

Extraction, i.e., the step prior to SPE purification, is quite simple for glyphosate in cereals. With glyphosate being a polar compound, the addition of water, acidified water, or methanol–water mixtures is used, followed by centrifugation. In an ‘Interlaboratory comparative study on the quantitative determination of glyphosate at low levels in wheat flour’, participants’ laboratories used water or acidified methanol or a methanol–water mixture [1]. Zhang et al., for the analysis of glyphosate in corn, used 10.0 mL of HPLC-grade water which was added to finely ground corn power [35], while Granby et al. analyzed glyphosate in cereals that were milled and ground to a particle size of 0.5 mm; then, 3 g was extracted twice with 25.0 mL MilliQ water [61]. Sorokin et al. used a similar extraction procedure for tea samples with 25 mL of deionized water acidified by 0.1 mL of 37% HCl [34].

#### 5.1.3. Advantages of Indirect Determination

Indirect determination means the use of derivatization. Regarding FMOC derivatization, it must be noted that originally, the determination of glyphosate and AMPA via liquid chromatography took place mainly with fluorimetry, and that in the absence of derivatization, the two molecules had practically no absorption (analysis not possible). A precolumn derivatization step with FMOC-Cl yielded highly fluorescent derivatives of the analytes [106]. Subsequently, even with the use of mass spectrometry, the standard ISO 16308:2014 has established the use of FMOC derivatization together with the triple quadrupole analyzer and a C18 column [104]. Derivatization is intended to improve at least one of the principal analytical parameters, namely, detection sensitivity or separation selectivity, by modifying the hydrophobic/hydrophilic character of the target molecule [107]. Glyphosate and AMPA are very small and polar compounds that lack chromophores or other heteroatoms that could facilitate their sensitive detection. In addition, the amphoteric nature of these agents makes their concentration and purification by normal-phase or reversed-phase SPE very difficult [108]. Obviously, from what has been said, the determination of glyphosate and AMPA with C18 columns works properly in indirect mode only.

#### 5.1.4. Direct Determination

Several works analyzing glyphosate via liquid chromatography do not make use of derivatization. Such methods use direct determination and work properly when columns different from the reversed-phase ones are used. We are talking about ion chromatography, HILIC, and Hypercarb columns.

Ion chromatography methods for glyphosate determination in cereals often require simple sample treatment consisting of homogenization, extraction, and centrifugation. Subsequently a purification step by filtration or SPE follows. Granby et al. analyzed glyphosate in cereals using an anion chromatography column coupled to a triple quadrupole IC-MS/MS system with ESI in negative-ion mode. They used NaHCO_3_ as a mobile phase together with a micro-membrane suppressor for removing Na^+^ ions since salts may harm the spectrometer. The transitions monitored (MRM mode) were 168 → 150 *m/z* and 168 → 124 *m/z* [61]. Similarly, Zoller et al. determined glyphosate and AMPA in cereals and other foodstuffs via an anion exchange method by using a triple quadrupole IC-MS/MS system with ESI in negative-ion mode [62].

HILIC (Hydrophilic Interaction Liquid Chromatography) is a suitable technique for separating polar compounds and is opposite to reversed-phase chromatography. Thus, it is suitable for underivatized glyphosate, which the reversed phase could not determine as it is. Ding et al. (2016) used an HILIC/WAX column (WAX = Weak Anion Exchange) to analyze glyphosate in plant-derived food by means of a triple quadrupole and ESI in negative ion mode. The column used was a Click TE-Cys (cysteine-based zwitterionic stationary phase). Samples such as soybean and corn were homogenized, extracted, centrifuged, and SPE-purified. The transitions monitored were 168 → 150 *m/z* and 168 → 63 *m/z*. To eliminate the matrix interference to the maximum extent, a two-step coupled SPE cartridge system was used. The matrix effect can lead to ion suppression, which is a well-known phenomenon for ESI-MS in LC. Ion suppression can also occur in ion chromatography, resulting from high buffer concentrations in the mobile phase; it leads to reduction in ionization efficiency with a subsequent negative effect on sensitivity. The limit of quantitation observed by Ding et al. was 0.02 mg kg^−1^ [109]. Similarly, Li et al. (2009) used the HILIC technique to analyze glyphosate in fruit and vegetables [110].

Thermo Scientific^TM^ Hypercarb^TM^ columns are porous, graphitic carbon columns suitable for the analysis of polar compounds by liquid chromatography without the need for derivatization. Chiarello et al. (2019) analyzed glyphosate and AMPA in edible oils using a Hypercarb^TM^ column with a 100 × 2.1 mm i.d. 5 µm particle size coupled to an LC-MS/MS triple quadrupole system in ESI negative-ion mode. The matrix effect observed was negligible; therefore, quantification was performed using solvent standard calibration and not matrix-matched calibration. In this particular case, such a negligible matrix effect was observed without the use of a cleaning step, but it could be necessary for grain samples. The transitions monitored were the same as those mentioned above: the fragmentation of the deprotonated molecular ion at *m/z* 168 of glyphosate yielded two product ions at *m/z* 150 and 63. The transitions monitored for AMPA were 110 → 63 and 110 → 79 [111]. Other works using the Hypercarb^TM^ column coupled to LC-MS/MS for analyzing glyphosate and AMPA exploit similar transitions [112].

#### 5.1.5. Glyphosate and Glufosinate

It is possible to observe that many of the research papers aimed at analytically determining glyphosate and AMPA include the compound glufosinate (2-amino-4-[hydroxy(methyl)phosphoryl]butanoic acid) in the determination. This is due to the similarity in the chemical structure of glyphosate and glufosinate, but there are no other links between them, although both can be used as herbicides. Glufosinate is a natural compound [113] that was first isolated from the bacteria *Streptomyces viridichromogenes* and *Streptomyces hygroscopicus* [114], while glyphosate was first discovered as a synthetic compound.

### 5.2. Gas Chromatography

Apart from liquid chromatography, the determination of glyphosate and AMPA can also be carried out with other instrumental techniques [103,115,116,117], among which gas chromatography stands out, with derivatization as a mandatory preliminary step. Initially, various types of detectors were used, such as FPD, NPD, and ECD [118,119,120]. Subsequently, the use of mass spectrometry (GC-MS) became increasingly established, although the simpler NPD and FPD detectors are still currently exploited in some cases [51,102].

#### 5.2.1. Sample Preparation and Perfluoroalcohol/TFAA Derivatization

**Figure 5:**
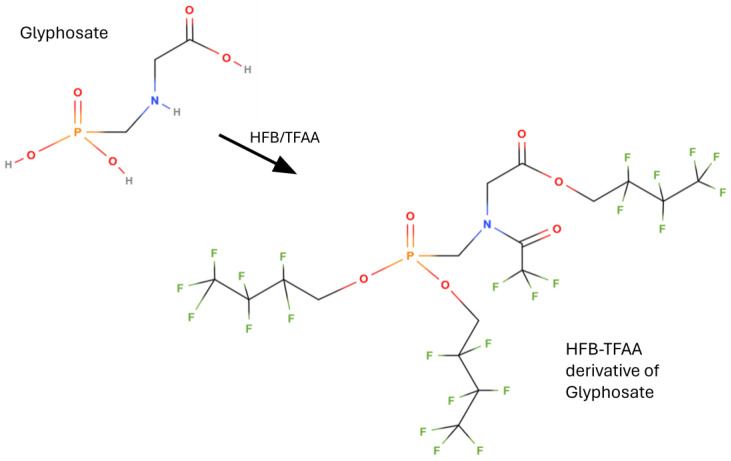
Derivatization of glyphosate with 2,2,3,3,4,4,4-heptafluoro-1-butanol (HFB) and trifluoroacetic anhydride (TFAA) for determination carried out by gas chromatography.

**Figure 6:**
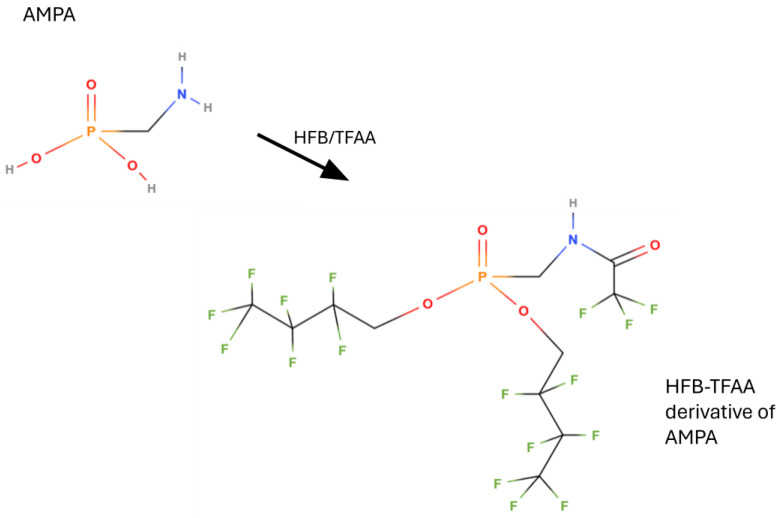
Derivatization of AMPA with 2,2,3,3,4,4,4-heptafluoro-1-butanol (HFB) and trifluoroacetic anhydride (TFAA) for determination carried out by gas chromatography.

From the first works to the present day, the largely prevalent derivatization for determining glyphosate in gas chromatography is that using perfluoroalcohols and perfluorinated anhydrides, with trifluoroacetic anhydride (TFAA) used almost universally. In this way, all phosphonic and carboxylic acid groups are esterified, and all amino groups are acylated, as shown in Figure 5 and Figure 6. Such derivatization was first applied by Deyrup et al. in 1985 [118]. This is also the procedure codified in the AOAC Official method 2000.05 ‘Determination of glyphosate and AMPA in crops, gas chromatography with mass-selective detection’ [121,122,123]. A variant of the derivatization in Figure 5 and Figure 6 is the use of 2,2,2-trifluoroethanol (TFE) instead of HFB [46,48,49,50,51]. The crops tested in the interlaboratory study supporting the acceptance of the AOAC method were field corn grain, soya forage, and walnut nutmeat, but it is applicable to a wide variety of crops and processed commodities: over 100 matrices were successfully analyzed with virtually no method modifications required.

Sample preparation involves the steps of extraction, purification, and derivatization. In summary, the extraction of grains requires homogenization with water, centrifugation, the addition of dichloromethane, a second centrifugation, the addition of an acid modifier, and a third round of centrifugation. The supernatant is so sent for a cation exchange cleanup.

The cleanup is achieved with a cationic exchange SPE column on which the supernatant from the previous step is loaded. Then, elution with a specific mobile phase is carried out.

Derivatization is carried out with HFB and TFAA kept at a low temperature until they are added to the eluate for the reaction, which takes place at 85–90 °C. After the addition of a citral reagent, the solution is ready for the gas chromatographic analysis.

#### 5.2.2. Instrumental Setup

The apparatus to be used is a gas chromatograph equipped with a capillary column (0.25 mm ID × 30 m, 0.50 µm film thickness) of cross-linked 95% methyl-5% phenyl silicone phase. Helium is used as a carrier gas at a flow rate of about 30 cm/s at 180 °C, equal to about 40–50 kPa (6–7 psi) at the column head. The suggested column oven temperature program is the following: an initial temperature of 90 °C, held for 1.5 min, increased to 300 °C at 30 °C/min (20 °C/min if limited by instrument capabilities), and held at 300 °C for 4 min. An alternative program for increased resolution is the following: an initial temperature of 60 °C, held for 1.5 min, increased to 120 °C at 10 °C/min, held at 120 °C for 1.0 min, increased to 300 °C at 30 °C/min, and held at 300 °C for 4 min. The injection port temperature is 200 °C, and the injection volume 2–5 µL in splitless mode.

The AOAC method requires that mass spectrometry is used as a detection technique by means of a quadrupole instrument, capable of providing electron impact mass spectra over an amu range up to *m/z* 650 operated in selected-ion monitoring (SIM) low-resolution mode. The method qualification data are obtained exclusively with quadrupole instrumentation only, but some participant laboratories in the development of the AOAC method have proposed the use of an ion trap as a viable alternative after some minor method modifications are applied. These included the elimination of the citral reagent in the final ethyl acetate extract, the use of a programmed variable-temperature GC inlet, and the use of methylene chloride as a keeper during the evaporation of the derivatization reagents [122]. MS/MS determinations are also possible. Royer et al. reported a successful ion-trap tandem MS application of the HFB/TFAA derivatization procedure to the analysis of glyphosate and AMPA in water, blackcurrants, and hazelnuts [47].

**Figure 7:**
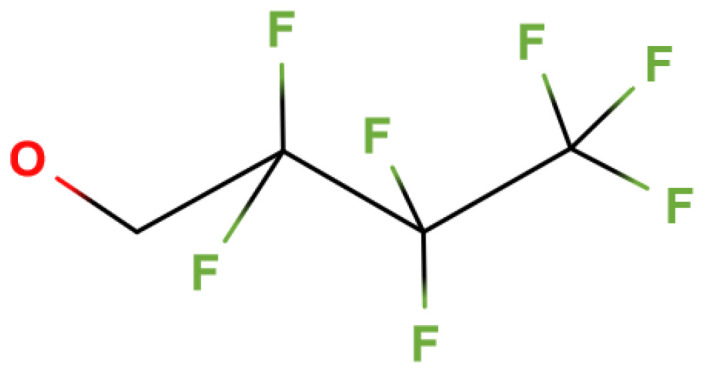
Fragment 2,2,3,3,4,4,4-heptafluoro-1-buthoxy- derived from the EI ionization of the glyphosate derivative in Figure 5.

The major ion fragments that can be used for the determination of glyphosate and AMPA derivatives via GC-MS, in SIM mode, are indicated in the AOAC method. They are 611.5, 584, and 460 *m/z* for glyphosate and 502, 446, and 372 *m/z* for AMPA. Although 611.5 and 446 *m/z* provide the greatest response for the glyphosate and AMPA derivatives, respectively, the alternative ions can be used for confirmatory analyses. The alternative ions may also be helpful for eliminating/reducing problematic interferences. The ion 611.5 *m/z* for the glyphosate derivative can be explained as follows. The molecular weight of the glyphosate derivative in Figure 5 is 811 amu. The need to monitor the ion 611.5 *m/z* after ionization results from the loss of an ion of about 199 *m/z* given that 811−611.5 = 199.5 *m/z*. The fragment lost is the group 2,2,3,3,4,4,4-heptafluoro-1-buthoxy-, as shown in Figure 7, which is indeed a fragment of about 199 *m/z*. The fragmentation mechanism is the same when 2,2,2-trifluoroethanol (TFE) is used in place of HFB, but in such a case, there is the loss of a 2,2,2-trifluoroethoxy- group (99 *m/z*). Therefore, with TFE, one of the main ions to be monitored for the glyphosate derivative (of 511 amu) is 412 *m/z*.

**Figure 8:**
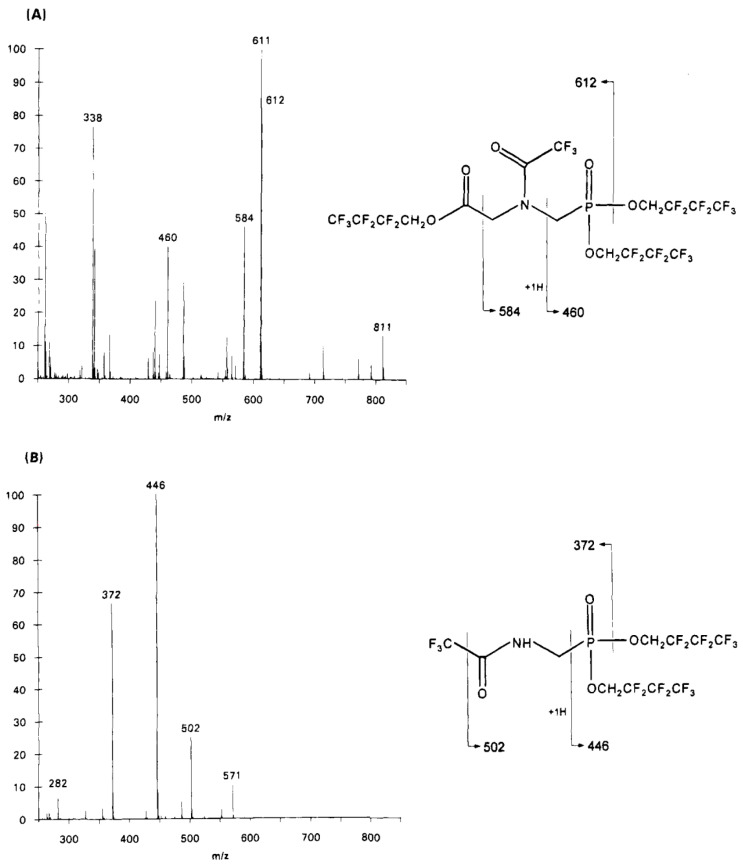
Electron impact mass spectra and structures of (**A**) glyphosate derivative (MW 811) and (**B**) AMPA derivative (MW 571). Reprinted with permission from Alferness and Iwata [123]. Copyright 1994 American Chemical Society.

Other ions of interest are those derived from the loss of a CF_3_ group (69 *m/z*), which, for the HFB derivative of AMPA (571 amu), results in a 502 *m/z* ion fragment, while for the TFE derivative of AMPA (371 amu), it yields a 302 *m/z* ion fragment [124]. Other relevant ions in SIM mode or transitions that are exploited in tandem mass spectrometry are available from the literature [43,44,45,46,47,48,49,50,51,52]. Figure 8 shows the mass spectra of glyphosate and AMPA when derivatized with heptafluorobutanol and trifluoroacetic anhydride, as obtained in the work of Alferness and Iwata [123].

#### 5.2.3. Alkylsilyl Derivatization

**Figure 9:**
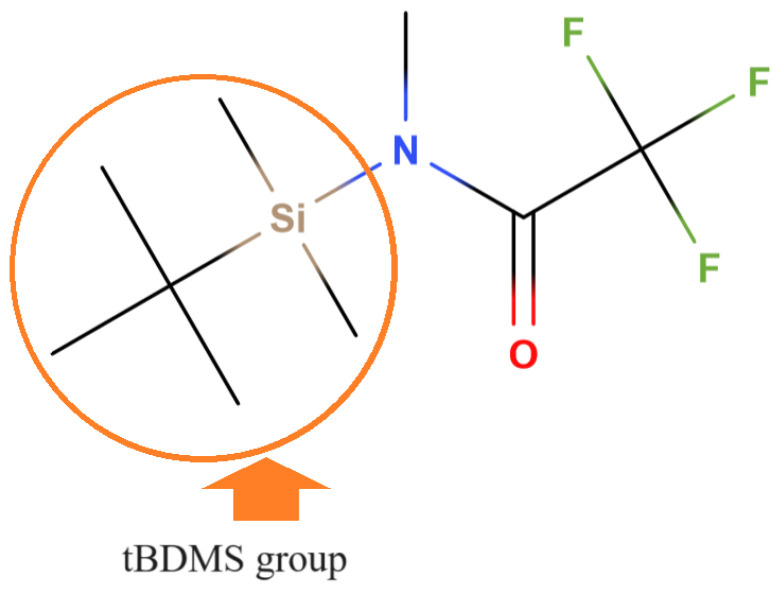
The derivatizing agent MTBSTFA. The tBDMS group is indicated in the circle; tBDMS stands for tert-butyldimethylsilyl-.

Alkylsilyl derivatization is a little-used procedure: it is mainly exploited in the gas chromatographic analysis of glyphosate and AMPA in biological fluids, such as serum and urine [125,126,127,128]. Furthermore, in some cases, the sensitivity is lower with respect derivatization with perfluoroalcohols and perfluorinated anhydrides. However, alkylsilyl derivatization is reported here for the sake of completeness. By far the most used reagent is N-methyl-N-(tert-butyldimethylsilyl)trifluoroacetamide (MTBSTFA), as shown in Figure 9. Such a reagent is capable of replacing three hydrogens of the glyphosate and AMPA molecules with three tBDMS groups.

The chemical reaction yields a glyphosate derivative of 511 amu and an AMPA derivative of 453 amu. As reported by Tsunoda [129], when MTBSTFA is used, the ions of interest to be monitored in GC-MS analysis are MW-15 and MW-57; therefore, the ions to be monitored are 496 and 454 *m/z* for glyphosate, while for AMPA, they are 438 and 396 *m/z* [126,127,129].

Other proposed alkylsilyl derivatizations involve the use of N,O-bis(trimethylsilyl)trifluoroacetamide (BSTFA) [130,131] or N-methyl-N-(trimethylsilyl)trifluoroacetamide (MSTFA) [132], which replace the hydrogens of glyphosate and AMPA with a trimethylsilyl group (TMS group).

### 5.3. High-Resolution Mass Spectrometry

The increasing availability of high-resolution mass spectrometers in analytical laboratories has led to the possibility of determining glyphosate in complex matrices in a more rapid and reliable way. Despite the higher purchase cost, there are some advantages to a high-resolution instrument compared to a low-resolution one. The ability to work at resolutions of up to 70,000 FWHM (Full Width at Half Maximum) for *m/z* values of 200 [133,134] allows us to monitor fragments up to four or five decimal places (exact masses). This involves the following:

(a) Simplifying sample preparation. Complex matrices may have interfering ion fragments with masses equal to those of the target analyte when measured at low resolution (unit resolution) and with the same chromatographic retention time. These are so-called isobaric interferences [135]. With low-resolution instruments (triple quadrupoles, ion traps), thorough purification of the sample is required to avoid this drawback. With high-resolution instruments, there is no such problem because the exact mass of the target analyte is monitored.
(b) Chromatographic runtimes. It is possible to shorten the chromatographic runs since possible coelutions of isobaric peaks do not lead to any inaccuracy if the exact mass is monitored. An extension of this approach is the Flow Injection technique, whose main feature is the injection of the predefined sample volume directly to the MS source, with no chromatographic separation [135].

**Figure 10:**
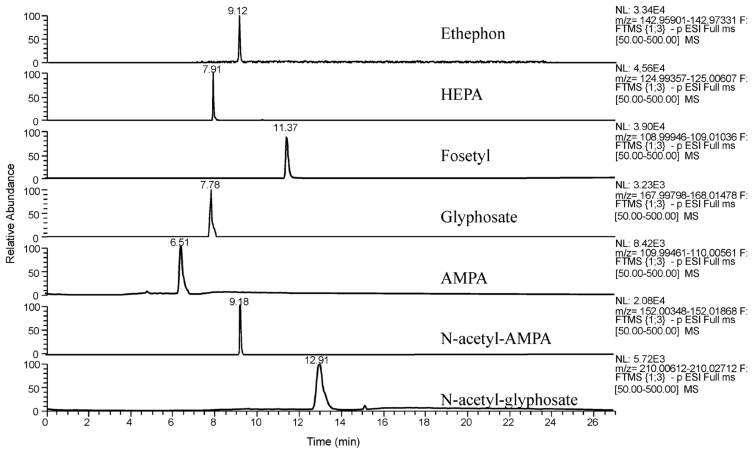
Extracted ion chromatograms from a spiked aubergine sample (100 µg kg^−1^) of the targeted compounds. Reprinted from Manzano-Sánchez et al., 2020 [137].

The high-resolution MS analysis of glyphosate in food matrices is mainly performed by liquid chromatography with the use of the Orbitrap apparatus that was introduced into mainstream MS in 2005 [136]. The LC-Orbitrap technique does not require derivatization. Rajski et al. analyzed glyphosate, AMPA, N-Acetyl glyphosate, N-Acetyl AMPA, and other highly polar pesticides in fruits and vegetables using Ion Chromatography-Q-Orbitrap with Electrospray Ionization in negative polarity [134]. The column used was a Dionex IonPac AS19, and elution was performed with KOH. The injection volume was 50 µL with an observed limit of quantitation of 0.01 mg kg^−1^ for all investigated pesticides. Sample preparation consisted of adding a water/methanol mixture to 10 g of sample, which was shaken and centrifuged and then diluted with water before the injection. The fragments monitored for glyphosate were 168.0067 *m/z*, 62.9637 *m/z* (quantifier ion), and 78.9588 *m/z* (qualifier ion), while for AMPA, they were 110.0012 *m/z* (quantifier ion), 62.9637 *m/z* (qualifier ion), and 78.9588 *m/z*. The fragments 168.0067 and 110.0012 *m/z* were derived from MS1 measurements, and the other fragments derived from MS2 (tandem) measurements. The retention times were 16 and 12 min for glyphosate and AMPA, respectively. Similarly, Manzano-Sánchez et al. determined glyphosate, AMPA, and other pesticides in fruits and vegetables by using the following system: UHPLC-Q-Orbitrap apparatus with a Torus DEA column with diethylamine as a stationary phase [137]. Elution was carried out with water (0.9% formic acid) and acidified acetonitrile (0.9% formic acid). Sample preparation was based on the QuPPe method (Quick Polar Pesticide method [138,139]) and involved the addition of water/acidified methanol to the sample that was homogenized and centrifuged, and then, 1 mL of the supernatant was filtered and injected. The ion fragments monitored for glyphosate were 168.00673, 78.95795, and 62.96304 *m/z*. The ion fragments monitored for AMPA were 110.00125, 78.95975, and 62.96304 *m/z*. The retention times were about 8 and 6 min for glyphosate and AMPA, respectively (Figure 10).

Other works used the Ion Chromatography-Q-Orbitrap system for monitoring glyphosate and AMPA in honey [140] or UHPLC-Q-Orbitrap with a Torus DEA column to analyze glyphosate and AMPA in drinking water [133]. In this last work, the fragment ions monitored for glyphosate were 168.00620 (precursor), 107.02619 (quantification; MS2, collision energy 25 V), and 133.00546 *m/z* (confirmation; MS2, collision energy 20 V). For AMPA, the following fragments were selected: 110.00043 (precursor), 62.96358 (quantification; MS2, collision energy 25 V), and 80.97415 *m/z* (confirmation; MS2, collision energy 20 V).

## 6. Conclusions

The European Union’s decision to allow the use of glyphosate came five months ago. This happened after a long controversy that lasted a few years and is still ongoing. This controversy reflects the state of the art: on the one hand, little is known about the toxicity of this herbicide; on the other hand, there is probably no valid alternative to guarantee food production that is adequate for global needs. Moreover, this does not consider the other uses of glyphosate, such as the maintenance of parks, gardens, roads, and railways, which are equally important. The present review addresses this topic by examining all points of the issue and delving into its chemical–analytical aspects, since what is currently needed is accurate information both on the toxicity of glyphosate and its presence in the food chain. The main outcome of this review is a discussion of the methods for the analysis of glyphosate in cereals and related matrices: the state of the art is presented without neglecting any detail necessary for researchers involved in this subject. The other outcomes are the reporting of reasoned opinions both for and against the toxicity of glyphosate, as well as the regulatory status, the fate, and finally, the accumulation of the herbicide in the environment and in the various parts of the cereal grain. Regarding this last point, it was demonstrated that most of the total glyphosate mass resides in the outer kernel layers, with higher concentrations in bread from whole-grain flour. Given the alleged health benefits of wholemeal flours, such a finding is expected to be relevant to future ‘benefit-risk ratio’ assessments.

## Figures and Tables

**Table 1:** Glyphosate and AMPA concentrations measured in cereals and related foods.

Cereals and Cereal-Based Foods	Glyphosate Concentration (mg kg^−1^)	AMPA Concentration (mg kg^−1^)	Reference
Barley	<0.45	n.a. ^1^	[61]
Oats	<0.08	n.a. ^1^	[61]
Rye	<0.04	n.a. ^1^	[61]
Durum wheat	0.421 (max.)	0.0247 (max.)	[62]
Wheat	<0.13	n.a. ^1^	[61]
Wheat	6.1–11.1	n.a. ^1^	[4]
Wheat bran	<0.7	n.a. ^1^	[61]
Wheat flour	0.02	n.a. ^1^	[61]
Bread	0.0458 (max.)	traces	[62]
Breakfast cereals	0.291 (max.)	0.01 (max.)	[62]
Flour and baking mixtures	0.133 (max.)	traces	[62]
GM soybean	0.4–8.8	0.7–10	[13]
GM corn	0.15	0.49	[63]
Wheat	0.373	0.034	[5]
Barley	2.15	0.041	[5]
Whole grain	0.0257	n.a. ^1^	[64]
White bread	0.0149	n.a. ^1^	[64]
Soy-based infant formulas	0.03–1.08	0.02–0.17	[65]
GM soybean	0.1–1.8	0.9 (max.)	[66]
Corn flour	0.0052–0.3 ^2^		[67]
Breakfast cereals	0.006–0.034	n.a. ^1^	[68]
Wheat flour	<0.03	n.a. ^1^	[69]
Wheat bran	1.62 (max.)	n.a. ^1^	[70]

^1^ not available. ^2^ sum of glyphosate and AMPA.

**Table 2:** Maximum residue levels (MRLs) for glyphosate in cereals as established by international organizations (mg kg^−1^). Values updated as of December 2023.

Cereals and Related Crops	European Union [93,94]	FAO/WHO Codex [94,95]	U.S. EPA ^1^ [96]	Health Canada [97]
Barley	20	30	30	10
Buckwheat	0.1	30	30	
Maize/corn grains	1	5	5	3
Millet	0.1	30	30	
Oats	20	30	30	15
Rice	0.1		0.1	
Rye	10	30	30	
Sorghum	20	30	30	
Soya beans	20	20	20	20
Wheat	10	30	30	5

^1^ MRLs are referred to as ‘Tolerances’ in U.S.
